# Projecting the Contribution of Assisted Reproductive Technology to Completed Cohort Fertility

**DOI:** 10.1007/s11113-023-09765-3

**Published:** 2023-02-10

**Authors:** Ester Lazzari, Michaela Potančoková, Tomáš Sobotka, Edith Gray, Georgina M. Chambers

**Affiliations:** 1 Department of Demography, University of Vienna (Wittgenstein Centre for Demography and Global Human Capital (IIASA, OeAW, University of Vienna)), Vienna, Austria; 2 International Institute for Applied Systems Analysis, Wittgenstein Centre for Demography and Global Human Capital (IIASA, OeAW, University of Vienna), Laxenburg, Austria; 3 School of Demography, Australian National University, Canberra, Australia; 4 National Perinatal Epidemiology and Statistics Unit (NPESU), Centre for Big Data Research in Health and School of Clinical Medicine, Faculty of Medicine and Health, University of New South Wales, Sydney, Australia; 5 Vienna Institute of Demography, Wittgenstein Centre for Demography and Global Human Capital (IIASA, OeAW, University of Vienna), Vienna, Austria

**Keywords:** Assisted reproductive technology, Cohort fertility, Fertility postponement, Fertility recuperation, Fertility projections, Australia

## Abstract

Assisted reproductive technology (ART) is increasingly influencing the fertility trends of high-income countries characterized by a pattern of delayed childbearing. However, research on the impact of ART on completed fertility is limited and the extent to which delayed births are realized later in life through ART is not well understood. This study uses data from Australian fertility clinics and national birth registries to project the contribution of ART for cohorts of women that have not yet completed their reproductive life and estimate the role played by ART in the fertility ‘recuperation’ process. Assuming that the increasing trends in ART success rates and treatment rates continue, the projection shows that the contribution of ART-conceived births to completed fertility will increase from 2.1% among women born in 1968 to 5.7% among women born in 1986. ART is projected to substantially affect the extent to which childbearing delay will be compensated at older ages, suggesting that its availability may become an important factor in helping women to achieve their reproductive plans later in life.

**Supplementary Information:**

The online version contains supplementary material available at 10.1007/s11113-023-09765-3.

## Introduction

In most high-income countries, childbearing has been increasingly postponed to later ages (Beaujouan, 2020; Mills et al., 2011). Delayed parenthood is associated with lower completed fertility and a higher chance of remaining permanently childless as reproductive capacity declines with age, especially among women, whose fecundity peaks in their early-20s and declines in their mid- to late-30s (Eijkemans et al., 2014). For example, Leridon’s simulation model (2004) shows that approximately 75% of women trying to conceive at age 30 will have a conception ending in a birth within one year, compared with only 66% at age 35 and 44% at age 40. With the increase in the proportion of couples experiencing age-related infertility, demand for assisted reproductive treatments has also increased.

Assisted reproductive technologies (ART), such as in vitro fertilization (IVF), where the embryo is created in the laboratory and transferred into the uterus in the hope of achieving a pregnancy, have spread rapidly, with more than 8 million ART-conceived babies being born worldwide as of 2018 (International Committee Monitoring ART and ESHRE 2018). ART has become a mainstream treatment for infertility and its contribution to total birth rates has been steadily increasing, representing the birth of up to 8% of children in some countries (Wyns et al., 2020). Yet, ART utilization rates widely vary across countries due to the existence of different regulations and health insurance schemes (Chambers et al., 2014; Präg & Mills, 2017).

Despite the continuous technological improvements, ART cannot fully compensate for the age-related decline in female reproductive performance because the effectiveness of ART also declines with age (Leridon, 2017; McCarter et al., 2021; Sartorius & Nieschlag, 2010). However, alternative treatment options such as oocyte cryopreservation (egg freezing) or the use of third-party eggs from younger donors are expanding women’s reproductive potential.

As people are having children at older ages, the contribution of ART gains relevance also for population policy. While several rationales justify the public financing of ART (Mladovsky & Sorenson, 2010), its potential positive impact on the fertility rate often assumes a central role. Assisted reproduction is now regularly included among the policy responses to low fertility rates (Gray et al., 2021; McCurry, 2020; Sobotka et al., 2019) and in some countries governments already reimburse ART with the specific aim to increase the fertility rate (Birenbaum-Carmeli, 2008; Blyth, Yee and Lee 2013; Kim, 2019). However, empirical evidence on how ART is influencing fertility trends is still limited.

Previous research in Europe and the United States has mostly focused on the impact of ART on overall fertility by adopting either a period (Habbema et al., 2009; Hoorens et al., 2007; Tierney, 2022) or cohort (Leridon, 2017; Leridon & Slama, 2008; Sobotka et al., 2008) approach, with most studies showing a rather modest contribution of 2–5%. Studies that have looked at age-specific ART fertility have found that the absolute contribution of ART to the TFR often peaks in the mid-30s, while the relative share of ART births increases with age (Burcin et al., 2020; Lazzari et al., 2021; Tierney, 2022). For example, Lazzari et al., (2021) found that increases in period fertility rates at late (40 +) reproductive ages in Australia were largely driven by the increasing use of ART.

Recent projections indicate that the future contribution of ART to Australian births will likely continue to increase because of the compositional shifts in the female population away from low and medium education towards higher levels of educational attainment, characterized by a pattern of delayed childbearing (Raymer et al., 2019). However, the contribution of ART to the completed fertility of future cohorts remains unexplored and its influence on contemporary demographic processes, such as fertility postponement and recuperation, has not yet been studied in Australia and in other developed societies.

This study has two broad aims. First, it seeks to quantify the contribution of ART to completed cohort fertility rate (CFR) in Australia. Our scenarios allow disentangling the impact of three distinct mechanisms affecting future trends in ART birth rates: women’s use of ART, ART success rates, and wider adoption of egg freezing and donor eggs among women of advanced reproductive age. Our scenarios are specified by single age and thus also account for the shifting number of women across reproductive ages, while the adoption of a cohort approach provides a better estimate of the impact of ART to fertility over time compared to period analyses. Our second aim is to investigate the role played by ART in the recovery of fertility. Looking at fertility from a life course perspective, delayed childbearing at younger ages (postponement) is followed by higher fertility at older ages (recuperation) (Frejka & Calot, 2001; Lesthaeghe, 2001). However, the realization of reproductive plans at older ages—and thus the extent of fertility recuperation—is hampered by increasing infertility (Leridon, 2004), with many couples facing involuntary childlessness. Thus, greater utilization of ART can support the number of births and fertility rates, especially past the age of 35.

Australia represents an illuminating case study among the group of low fertility countries as it has one of the highest ART utilization rates in the world (Chambers et al., 2021), partly explained by the relatively supportive funding arrangement through Australia’s universal healthcare system, Medicare. The scheme covers approximately two-thirds of total costs for an unlimited number of ART cycles, with no restrictions based on age, number of cycles, or parity—limitations commonly in place in other countries (Allan et al., 2019). This absence of an age limit for publicly funded access to infertility treatment distinguishes Australia from most other countries and places it in a unique position to investigate the contribution of ART to fertility at advanced reproductive ages.

## Fertility Trends and Late Childbearing Desires in Australia

Completed cohort fertility of successive generations of Australian women has been steadily declining to an average of 2.0 children among women born in 1967–1971 (Lazzari, 2021). Such decline was accompanied by a rise in permanent childlessness, from 9.6% for women born in 1950 to 15.2% for women born in 1968,^1^ and by a continuous increase in the mean age at childbearing from 25.4 in 1971 (Australian Bureau of Statistics, 2001) to 30.6 in 2017 (Australian Institute of Health and Welfare, 2019a), mainly driven by the postponement of first births. Since the beginning of the 1980s, the prevalence of late (40 +) births also shifted from 1.2% to 4.8% in 2017. Similar changes occurred in other low fertility countries (Beaujouan, 2020; Billari et al., 2007). Moreover, repartnering has become common among divorced and separated women (Gray, 2015), which further contributes to the rising fertility at later reproductive ages.

**Fig. 1:**
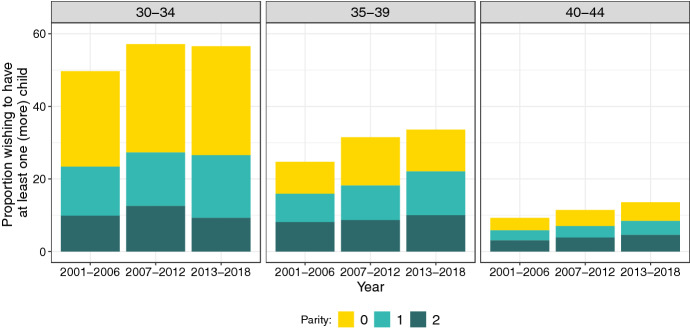
Share of women wishing to have at least one (more) child by age group and parity, Australia, 2001–2018. Source: Authors’ computations based on Household Income and Labour Dynamics in Australia (HILDA) survey, waves 1–18, release 18 (weighted)

Shifts in the timing of fertility are also reflected in recent surveys of reproductive intentions, with rising shares of Australian women in their 30s or 40s wishing to have a child in the future. Figure 1 illustrates the increase in the proportion of women wishing to have at least one (more) child between 2001 and 2016. This trend is more pronounced among women aged 35–39 (from 24.7% to 33.6%) and 40–44 (from 9.3% to 13.6%). Desiring more children is closely associated with being childless or having one child, although, among women aged 35 and above, desires seem to be growing even for those with two or more children. The distinction between compositional factors (age and parity) is essential for interpreting trends in childbearing desires and to understand individual motivations behind the increasing demand for ART as women typically resort to these technologies after their mid-30 s, especially to have a first child (Lazzari et al., 2021).

Taken together, the increase in childbearing desires at advanced reproductive ages and the age-related decline in the biological capacity to reproduce suggest that assisted reproduction is likely to become more important for future fertility trends. Indeed, ART can help reducing the gap between desired and achieved number of births as it provides an opportunity for women, men, and couples to partly reconcile the mismatch between the desired and biologically optimal age to have children. Arguably, the increase in the prevalence of late births and rising use of reproductive technologies are linked (Billari et al., 2007), and ART may partly support fertility at older ages by helping women to fulfill their reproductive desires at an age when many of them experience infertility.

## Materials and Methods

**Fig. 2:**
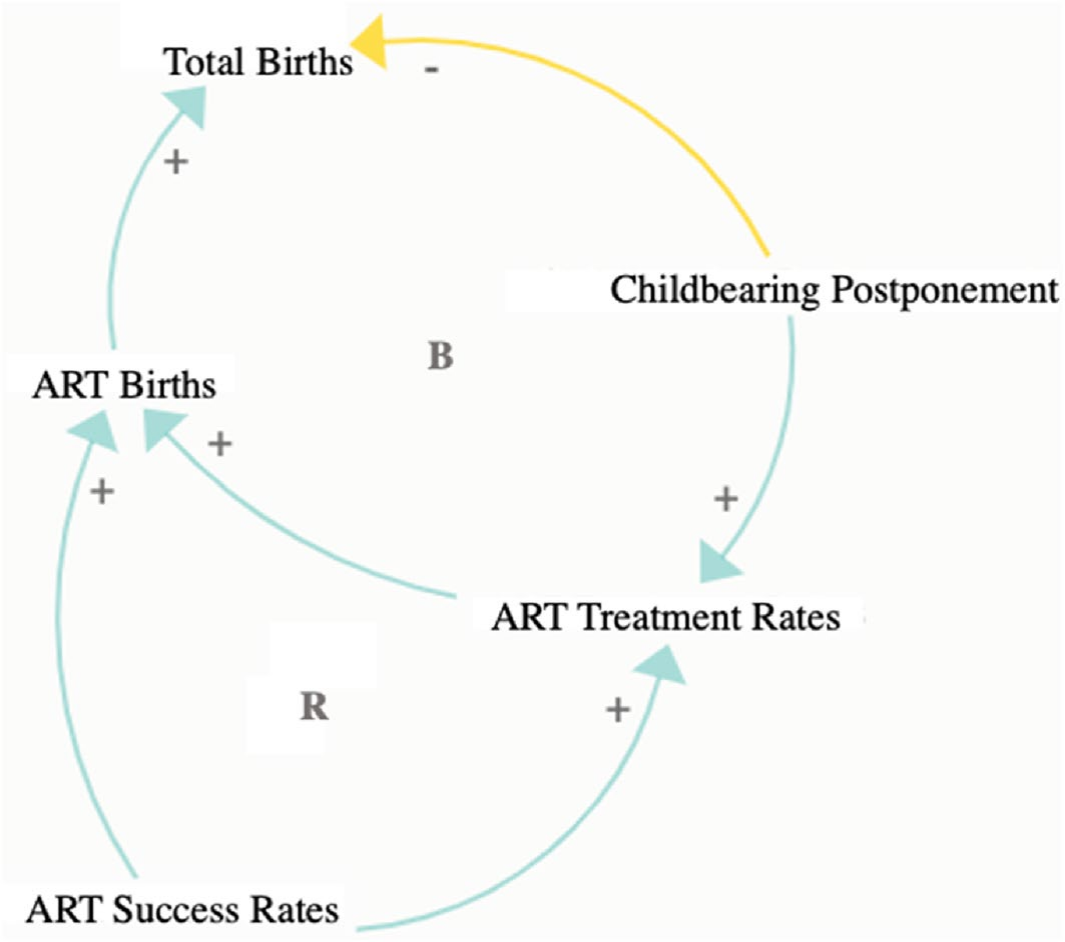
Causal loop diagram showing the contribution of ART treatment rates, ART success rates and childbearing postponement to ART births and to the total number of births. A balancing loop (B) stabilizes a component of the system while a reinforcing loop (R) reinforces such component. The “ + ” sign indicates a positive relationship between variables, while the “ − “ sign indicates a negative relationship between variables

To predict the future contribution of ART to CFR, the model brings together information on three distinct factors that influence this contribution: (1) cohort postponement of childbearing to later ages when the risk of experiencing infertility is higher; (2) changes in the uptake of ART treatments; and (3) changes in ART success rates. Our data do not allow us to distinguish each individual treatment. Rather, we analyze the share of women receiving at least one treatment at each age and, in turn, the share of women at each age having an ART-conceived child after receiving one or more treatments (success rate). Dependencies between these three components and their impact on ART births and total births are shown in the causal loop diagram in Fig. 2.

The total number of ART births is directly influenced by ART treatment and success rates and indirectly shaped by further childbearing postponement, which leads to a higher proportion of women seeking to have children at older ages when their reproductive potential diminishes. Figure 2 also shows links between treatment and success rates: an increase in the effectiveness of treatments may encourage higher utilization, while an increase in the use of ART may further support advances in the technology. The main outcome variable, the total number of births, is lowered by childbearing postponement, although ART births can partly compensate for such decline.

### Data Sources

The total number of ART births and treatments by age performed in Australia between 2010 and 2017 are sourced from the Australia and New Zealand Assisted Reproduction Database (ANZARD), a clinical registry that collects information about all ART treatments performed in Australia and the resulting pregnancies and birth outcomes (Newman et al., 2021). Since all Australian fertility clinics are required to report ART birth outcome information to ANZARD as part of their licensing requirements, full registration of ART births can be assumed. To supplement these data, we collected information on the total annual number of ART babies born to Australian women between 1998 and 2009 from the annual ANZARD reports. ART age-specific fertility rates for the period 1998–2009 are estimated by combining these absolute numbers with the relative ART age-specific fertility profile as observed in 2010. In combination, we were able to reconstruct and estimate ART treatments and births over a twenty-year period, 1998–2017. Fertility treatments that do not involve fertilization outside of the woman’s body, such as intrauterine insemination and ovulation induction, are not included because only births resulting from treatments involving ART are reported in ANZARD. Population birth data are drawn from the national birth registries (Australian Bureau of Statistics 2021a). The number of non-assisted births (conceived without ART) is obtained by subtracting the number of ART-conceived births from the number of total births in each year and by single age of mother.

The study takes a cohort perspective and focuses on women born in 1968–1986. The fertility of the cohort born in 1968 is complete and known, as these women reached age 49 in 2017, while projections are needed for later cohorts. All data on total and ART-related fertility occurring in 2018 and later are projected. The Lexis diagram displayed in Fig. S1 in section A of the Online Appendix material provides a visual representation of the data sources and analyzed cohorts.

### Modelling the Role of ART on Completed Fertility

The completed fertility rate, or ${CFR}_{c}\left(x\right)$, of a cohort of women born in year $c$ up to age $x$ is computed as the sum of their age-specific fertility rates as:

1 $$CFR_{c} = \mathop \sum \limits_{x = 15}^{49} f_{c} \left( x \right) = \mathop \sum \limits_{x = 15}^{49} \frac{{B_{c} \left( x \right)}}{{W_{c} \left( x \right)}}$$

where ${B}_{c}(x)$ is the number of births to women at age $x$ born in year $c$, and ${W}_{c}(x)$ is the number of women in the population at age $x$ born in year $c.$ In order to study how ART influences the ${CFR}_{c}\left(x\right)$, Eq. (1) is reformulated as the sum of ART and non-ART age-specific fertility rates, as:

2 $$CFR_{c} = \mathop \sum \limits_{x} \left( {f_{c}^{ART} \left( x \right) + f_{c}^{N} \left( x \right)} \right) = \mathop \sum \limits_{x = 15}^{49} \left( {\frac{{B_{c}^{ART} \left( x \right)}}{{W_{c} \left( x \right)}} + \frac{{B_{c}^{N} \left( x \right)}}{{W_{c} \left( x \right)}}} \right)$$

where ${B}_{c}^{ART}\left(x\right)$ indicates births conceived through ART and ${B}_{c}^{N}$ indicates births conceived without ART. ART age-specific fertility rates can be further decomposed as follows:

3 $$\mathop \sum \limits_{x} f_{c}^{ART} \left( x \right) = \mathop \sum \limits_{x} t_{c} \left( x \right) s_{c} \left( x \right)$$

where

4 $$t_{c} \left( x \right) = \mathop \sum \limits_{x = 15}^{49} \frac{{T_{c}^{ART} \left( x \right)}}{{W_{c} \left( x \right)}}$$

corresponds to the ART age-specific treatment rate (the proportion of women of age $x$ born in year $c$ receiving ART treatment out of all women of age $x$ born in year $c$ in the population) and

5 $$s_{c} \left( x \right) = \mathop \sum \limits_{x = 15}^{49} \frac{{B_{c}^{ART} \left( x \right)}}{{T_{c}^{ART} \left( x \right)}}$$

corresponds to the *ART* age-specific success rate (the number of ART-conceived babies born to women of age $x$ and born in year $c$ out of all women of age $x$ born in year $c$ receiving ART treatment).

The decomposition in Eq. (3) is suitable for the purpose of analysing the role of a change in the demand for reproductive treatments and success rates in determining the contribution of ART to age-specific fertility rates. This way of measuring success rates differs from the medical literature, where success is typically computed as the ratio of the number of treatment cycles to live births. Instead, the method used in this study reflects the age-specific probability that a woman undergoing ART treatment in a given year will be successful in having a desired birth.

### Cohort Fertility Forecasting

The forecasting of non-ART age-specific fertility rates is accomplished by using the linear five-year extrapolation method by Myrskylä et al., (2013). Inspired by the Lee-Carter model for period mortality (Lee & Carter, 1992) and Lee’s model for period fertility (Lee, 1993), this method is designed to estimate incomplete cohort fertility. The method extrapolates the trend in period age-specific fertility rates five years into the future based on the trend observed over the past five years, and then freezes the rates (Myrskylä et al., 2013) (the details of the method are provided in section B of the Online Appendix). In our study, this corresponds to forecasting period fertility rates from 2017 (the most recently observed year) to 2022.

In a recent assessment of twenty methodologies to forecast CFR, the five-year extrapolation method has been evaluated as the most accurate and outperforming more sophisticated forecasting models (Bohk-Ewald et al., 2018). This method is also preferred to using unlimited linear extrapolation, or the simpler freeze-rate approach, which assumes that the forecasted age-specific fertility rates will stay the same as in the most recent year, thus underestimating potential fertility recovery at higher ages when fertility timing is changing.

### Projection Scenarios of ART Fertility Rates

Five scenarios of ART fertility rates are formulated, based on different assumptions regarding future ART success rates and treatment rates (in Fig. 4). They are combined with the forecasted non-ART age-specific fertility rates, resulting in five alternative trajectories of future completed fertility accounting for the contribution of ART.^2^

1. *No-change scenario* (S1) assumes no further improvement in success rates and no further increase in treatment rates. Success and treatment rates remain fixed at their level observed in 2017.
2. *Extrapolated success rates scenario* (S2) assumes improving success rates combined with an unchanged use of ART. Success rates are extrapolated five years into the future, from 2018 to 2022, based on the five previous years, and then kept fixed at their latest extrapolated level. Treatment rates remain fixed at the level observed in 2017. This means that further increases in the number of ART births are solely due to improvements in the technology.
3. *Extrapolated treatment rates* scenario (S3) explores the impact of increasing demand for ART among cohorts as they age in combination with stable success rates (fixed at the level observed in 2017). Treatment rates are extrapolated five years into the future, from 2018 to 2022, based on the five previous years and are then kept fixed at their latest extrapolated level. This means that further increases in the number of ART births are solely due to increased utilization of treatments.
4. In the *Extrapolated success and treatment rates scenario* (S4) both success and treatment rates are extrapolated up to 2022 and then kept fixed at their 2022 level.
5. *Egg donation and freezing scenario* (S5) assumes high ART success rates for women over 40 due to increased use of donor eggs and/or frozen eggs. The practice of using donor eggs is most common among women above the age of 45, explaining the relatively high success rates at these ages. In this scenario, we compute the average success rates at age 46–49 for the period 2018–2022 and apply it to all women aged 40 and above. Our extrapolated ART success rates below age 40 remain the same as in scenarios 2 and 4, while extrapolated treatment rates are identical to scenarios 3 and 4.

While scenarios 2 and 3 are useful to disentangle the effect of an increase in ART treatment and success rates, it is unlikely that one will occur without the other. Hence, we consider the *Extrapolated success and treatment rates scenari*o (S4) as the most plausible scenario, reflecting the likely improvement in both ART treatment and success rates. The *No-change scenario* (S1) should be seen as a benchmark to evaluate the impact of projected changes compared to a situation without further increases in treatment rates and improvements in success rates. The *Egg donation and freezing scenario* (S5) illustrates a hypothetical situation where all women aged 40 and above would suddenly shift from using own fresh eggs to using frozen or third-party donor eggs after 2017. It provides insights into the potential future contribution of ART to fertility at late reproductive ages, should egg freezing become more widespread. While supply constraints, cost, legislation, and ethical concerns may prevent wider spread of donor eggs (Gleicher et al., 2020), recent evidence suggests a dramatic increase in the utilization of egg freezing in Australia (Johnston et al., 2021). This suggests that as more women are choosing to freeze their eggs at younger ages, which are closer to the biologically optimal age (Human Fertilisation and Embryology Authority 2018; Johnston et al., 2021), the use of oocyte cryopreservation may become increasingly important in influencing future fertility trends.

Australian research has linked the increasing demand for fertility treatment with educational change (Raymer et al., 2019), as highly educated women are more likely to delay family formation and, hence, to experience infertility. Moreover, access to ART care in Australia markedly differs across socio-economic groups (Lazzari et al., 2022). Between the cohorts of women born in 1968 and 1986, there has been a substantial educational expansion, with the proportion of tertiary educated women increasing from below 30% to 45% (Australian Bureau of Statistics 2021b). Our modelling strategy has indirectly incorporated the effect of this compositional shift in the scenarios assuming a rising trend in ART usage. As a robustness check, we evaluated how our results would change if we explicitly included educational attainment in the projection model (see section C in the Online Appendix). We found no substantial differences to the results presented in the next section.

### Projection Scenarios of ART Contribution to Fertility Recuperation

This study uses and builds upon the benchmark model of fertility postponement and recuperation (Frejka & Calot, 2001; Lesthaeghe, 2001) to investigate the impact of ART to these processes.

Fertility postponement and recuperation (recovery) can be measured for each cohort of interest in comparison with an older (reference) cohort. In the postponement phase, the cumulative completed fertility rate of successive cohorts is lower than that of the reference cohort because fertility at younger ages is declining, while in the recuperation phase, the cumulative difference gradually decreases as fertility at older ages is increasing relative to the reference cohort. If part of the “postponed” fertility is not recuperated later in life, it translates into a permanent decline in CFR across cohorts.

Using a benchmark cohort of interest, this framework allows a clear comparison of what proportion of postponed fertility has been recuperated or foregone relative to that cohort. The method is also particularly suitable for investigating how these proportions of recuperated or foregone fertility rates would change under different circumstances affecting fertility recuperation. For instance, the availability of ART partly relaxes reproductive constraints at older ages, allowing for higher recuperation levels as compared to a situation where ART is not available. Such contribution can be computed by first quantifying how much of the observed decline in cohort fertility below a certain age *m* is projected to be recovered after that age, relative to the reference cohort, and then by quantifying the proportion of such recuperation that is attributable to ART.

**Fig. 3:**
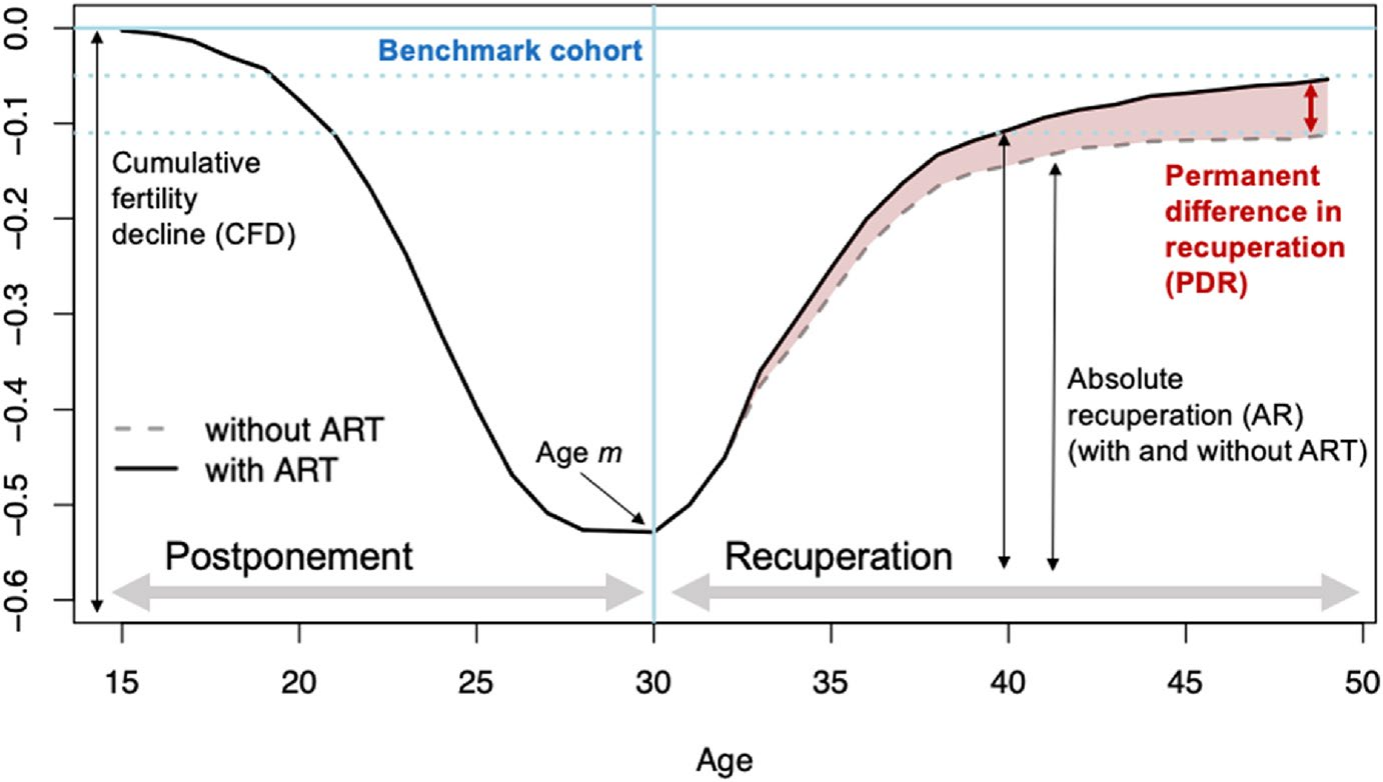
A simplified scheme of postponement and recuperation, indicating the potential contribution of ART. The design is inspired by Sobotka et al., 2012 (Fig. 1, p. 422) and adapted by the authors to show how accessibility to ART affects the demographic processes of fertility postponement and recuperation

Figure 3 shows how would these processes change with and without the availability of ART treatments. The cumulative fertility falls relative to the reference cohort until reaching a maximum difference at age *m*, which corresponds to the age of 30 for the cohorts analysed in this study, after which the fertility “gap” between these two cohorts starts narrowing.

The absolute increase in cumulated cohort fertility between age *m* and the end of the reproductive life at age 49—as compared to the reference cohort—is a measure of absolute recovery. Figure 3 illustrates that the availability of ART contributes to this recovery and shows how this model can be used to compare different scenarios of ART uptake and success rates and to investigate the differences in fertility recuperation levels associated with these scenarios.

### Results

#### Trends in Age-Specific Treatment and Success Rates

**Fig. 4:**
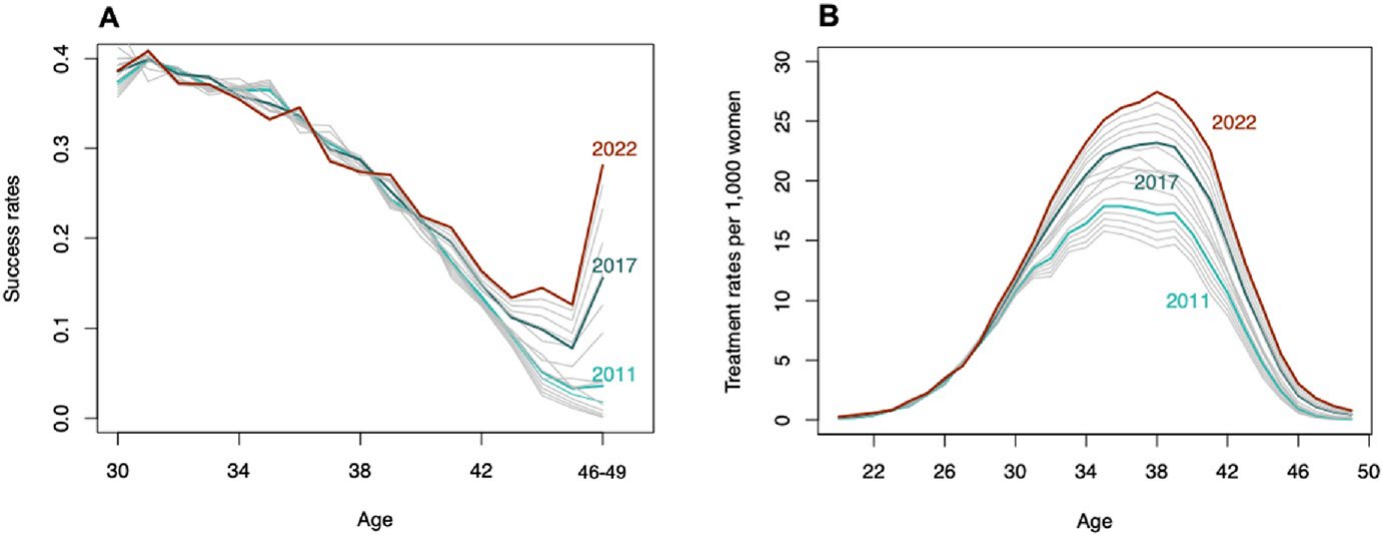
Estimated (2006–2009), observed (2010–2017) and projected (2018–2022) ART age-specific success rates **A** and ART age-specific treatment rates **B**. Due to the small number of ART births after age 45, the data for computing success rates at age 46–49 are aggregated. Results are obtained using Eq. (3). Source: Authors’ computations based on ANZARD and ABS data

Estimated (2006–2009), observed (2010–2017), and projected (2018–2022) trends in ART success rates and treatment rates used in the projection model are displayed in Fig. 4. Between the ages of 30 and 45, success rates gradually decline: while approximately 40% of treated women in their early 30s can expect to give birth to an ART-conceived child, by the age of 45, this proportion dramatically drops (to 5% over the period 2011–2017, on average). Since 2011, however, there has been a rebound in success rates at age 45 and above. For example, while only 3.6% of women receiving ART treatment at age 46–49 gave birth in 2011, this proportion jumped to 15.6% in 2017 and it is projected to reach 28% in 2022. The projected increase in success rates after 2017 reflects the ongoing shifts away from more “traditional” ART treatments to oocyte cryopreservation and ART cycles using donor eggs (Newman et al., 2021).

The distribution of age-specific treatment rates (Fig. 4B) resembles that of total age-specific fertility rates, although with an older age profile. The mean age at treatment is projected to increase from 35.6 in 2006 to 36.4 in 2022. Over the period of analysis, treatment rates per 1000 women aged 35–40 are projected to increase by 44%, from an average of 18 in 2006 to 26 in 2022.

#### Projected Impact of ART on Completed Cohort Fertility

When contrasting the *No-change scenario* (S1) with a hypothetical situation where no ART treatment is available, the projection suggests that there will be a slight decline in CFR and that women born in 1986 will have 1.9 children, on average (see Fig. S4 in section D of the Online Appendix). This is an all-time low in Australia, although still above the ‘very-low’ cohort fertility threshold suggested at 1.75 (Zeman et al., 2018).

The decline in CFR would be sharper in the absence of infertility treatment. For instance, without births resulting from ART, the CFR of the 1986 cohort would reach 1.8, approximately 5% (or 0.1 children per woman) less than its total projected value. The contribution of ART is likely to increase over time, growing in importance for women that are currently around age 30 or younger.

#### Differences Between Scenarios

**Fig. 5:**
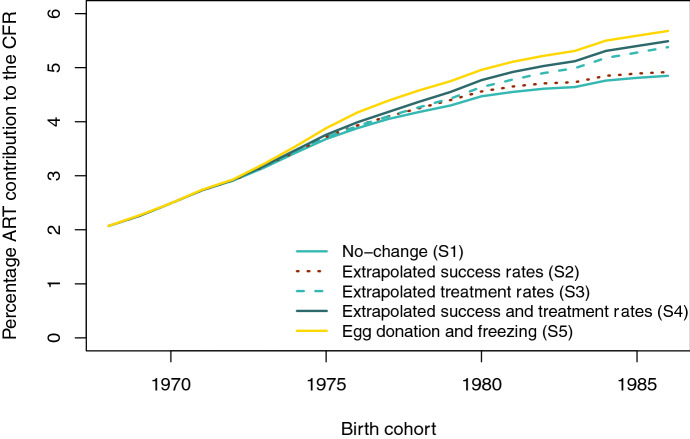
Observed (1968) and projected (1969–1986) percentage ART contribution to CFR. See section “Projection scenarios of ART fertility rates” for a detailed description of each scenario’s assumptions. Source: Authors’ computations based on ANZARD and ABS data

Figure 5 presents the projected relative contribution of ART use to CFR according to five alternative scenarios (the underlying numbers are shown in Table S3 in section D of the Online Appendix). All scenarios indicate a rising impact of ART on completed fertility, with the contribution of ART births to CFR more than doubling from 2.1% among women born in 1968 to 4.7–5.7% among women born in 1986. The impact of different scenarios becomes more distinct among women born in the late 1970s, who were around age 40 at the projection baseline. The slowest growth (to 4.7–4.8%) is projected by the *No-change scenario* (S1), which assumes no further change in success and treatment rates after 2017, and in the *Extrapolated success rates scenario* (S2). The *Extrapolated treatment rates scenario* (S3) shows a faster rise to 5.2% for the 1986 cohort, suggesting that increasing treatment rates may contribute more to future fertility than rising success rates of the treatments unless a significant improvement in technology brings about a faster improvement in success rates. This possibility is envisaged in the *Egg donation and freezing scenario* (S5), which projects greater contribution to CFR (almost 6% for the 1986 birth cohort). Although such a quick improvement in success rates at later ages is unlikely to materialize, this scenario sheds light on the potential impact of a fast adoption of egg freezing on late fertility.

#### Age-Specific Projections

**Fig. 6:**
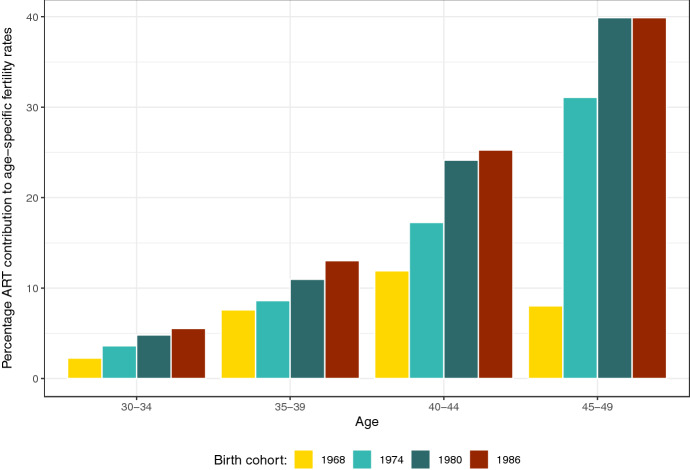
Observed (1968) and projected (1969, 1974, 1980, 1986) percentage ART contribution to age-specific fertility rates. Projected values are obtained using the extrapolated success and treatment rates Scenario (S4). Source: Authors’ computations based on ANZARD and ABS data

When the share of ART births out of total births is broken down by age group and single birth cohort (Fig. 6), the results show that if both success and treatment rates continue to increase, as hypothesized in the *Extrapolated success and treatment rates scenario* (S4), the probability of having a child conceived through ART increases with age and over cohorts. For instance, the percentage ART contribution to fertility rates among women aged 40–44 increases from 11.9% in the 1968 cohort to 25.3% in the 1986 cohort and the increase is even steeper, from 8.0% to almost 40%, at age 45–49.

However, at these advanced reproductive ages, the total number of births is relatively low as most childbearing has taken place before age 40. Since ART has a negligeable impact on births at younger ages (below 30), the overall contribution of ART to fertility in Australia remains much lower, approximately 5 percent, than the observed impact at age 40 and above. Yet, our findings suggest that the increasing contribution to the CFR of fertility rates at older reproductive ages will be strongly supported by the increasing availability and use of ART. A smaller rise in ART births at later ages is obtained using the *Extrapolated treatment rates scenario* (S3) (not shown), with the percentage ART contribution to fertility rates increasing up to 23.0% for women aged 40–44 and 21.1% for women aged 45–49 born in 1986.

#### Projected Impact of ART on Fertility Recuperation

**Fig. 7:**
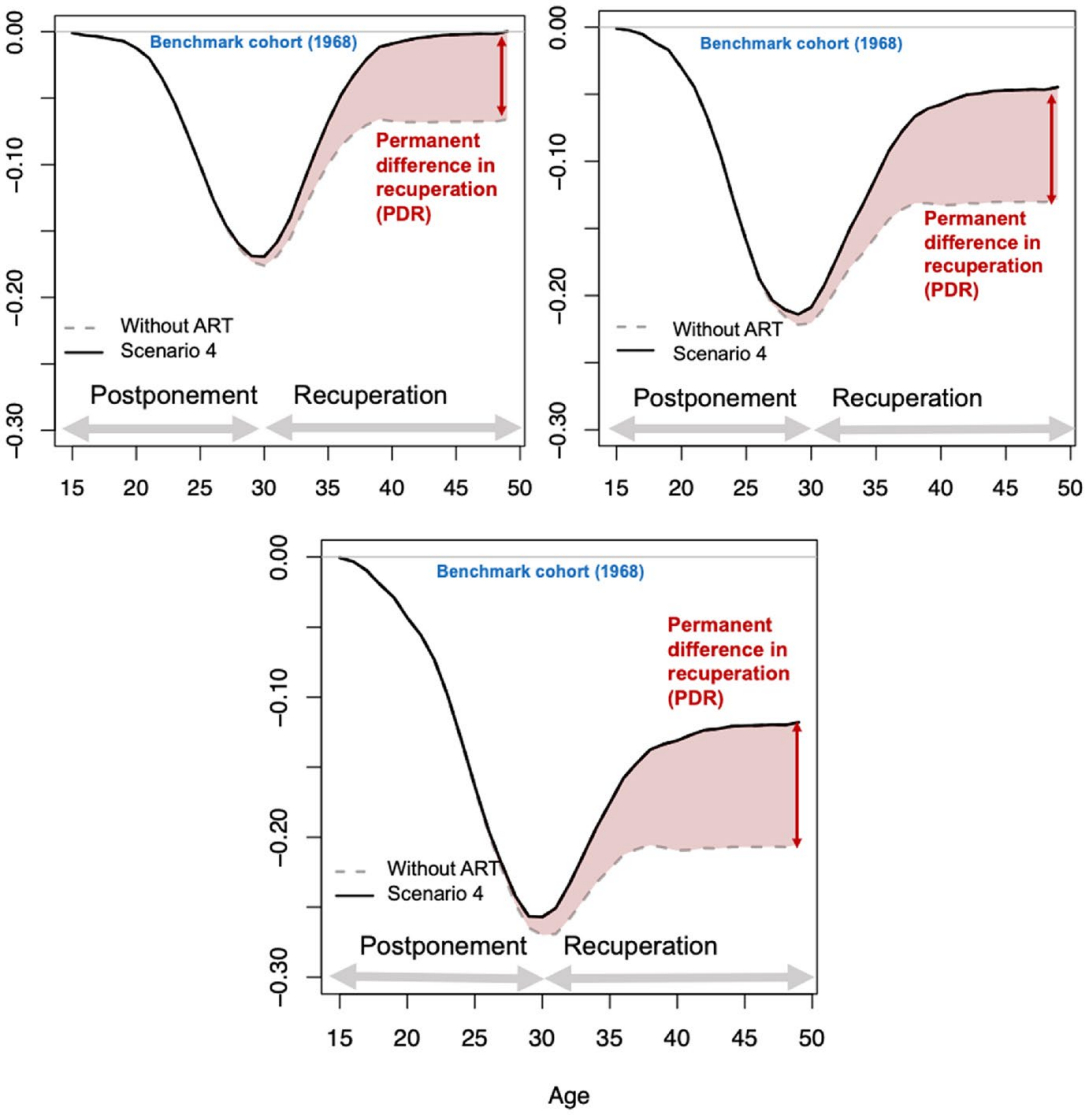
Projected cumulated cohort fertility of women born in 1974, 1980, and 1986 compared to the observed reference cohort (born in 1968), Scenario 4. Completed fertility of the reference cohort does not include ART births. Source: Authors’ computations based on ANZARD and ABS data. Source: Authors’ computations based on ANZARD and ABS data

Figure 7 illustrates the projected dynamics of the postponement and recuperation processes for three selected cohorts (1974, 1980, and 1986), as compared to the reference cohort born in 1968. Supplementary information is provided in Table 1. With age, ART plays an increasingly important role in offsetting the decline in CFR. A partial recovery of births between the ages of 30 and 40 occurs, regardless of ART. However, past the age of 40, the increase in ART-conceived births becomes the only driver of further fertility recuperation. The absolute difference in recuperation between a hypothetical situation in which ART is not available and the *Extrapolated success and treatment rates scenario* (S4) increases over cohorts, from 0.06 for the 1974 cohort to 0.09 for the 1986 cohort (Table 1). This pattern reflects the greater reliance of women and couples on ART in fulfilling their childbearing desires at advanced reproductive ages and the increasing demand for ART as a family building option across generations.

**Table 1:** (a) Projected decline in completed fertility by age 30 for cohorts born in 1980, 1982, 1984, and 1986, with and without ART; (b) Projected recuperation in completed cohort fertility for cohorts born in 1980, 1982, 1984, and 1986, with and without ART; (c) Permanent difference in recuperation

	Birth cohort		
Year of birth Age at forecast in 2017	1974 35	1980 33	1986 31
(a) Projected cumulated fertility decline (CFD) in CFR by age 30*			
Without ART	0.176	0.221	0.270
With ART (all scenarios)	0.169	0.209	0.257
(b) Projected absolute recuperation (AR) above age 30*			
Without ART (%)	0.110 (62.5)	0.092 (41.7)	0.065 (24.0)
No-change Scenario (S1) (%)	0.169 (100)	0.164 (78.5)	0.139 (54.1)
Extrapolated success and treatment rates Scenario (S4) (%)	0.171 (100)	0.170 (81.2)	0.151 (58.6)
Egg donation and freezing Scenario (S5) (%)	0.172 (100)	0.173 (83.0)	0.154 (59.9)
(c) Permanent difference in recuperation (PDR)			
No-change Scenario (S1)	0.059	0.072	0.074
Extrapolated success and treatment rates Scenario (S4)	0.060	0.078	0.086
Egg donation and freezing Scenario (S5)	0.074	0.086	0.089

^*^As compared to the completed fertility of the benchmark cohort (1968), non-ART births only

See section “Projection scenarios of ART fertility rates” for a detailed description of each scenario’s assumptions. Source: Authors’ computations based on ANZARD and ABS data

In the 1974 cohort, fertility recovery after age 30 is complete when considering ART births. Among women born in 1980, 81.2% of births delayed before age 30 (compared to the 1968 reference cohort) would eventually be realized, according to the *Extrapolated success and treatment rates scenario* (S4). The projected recuperation is 78.5% under the *No-change scenario* (S1) and 83% under the *Egg donation and freezing scenario* (S5). In the youngest cohort born in 1986, recuperation is projected to drop to 58.6% under S4, with approximately half of such recuperation attributable to ART. Hence, while the use of ART substantially contributes to women having births at older reproductive ages, it will not fully compensate for the decline in CFR.

#### Study Limitations

When estimating the potential impact of ART on CFR, modelling assumptions and potential limitations should be acknowledged. First, this study assumes that births conceived through ART would not have happened without the treatment. However, some couples having an ART birth would have eventually conceived spontaneously (Cahill et al., 2005; de La Rochebrochard et al., 2009; Troude et al., 2012), leading to an overestimation of the actual impact of ART on fertility levels.

Second, the availability of ART may trigger unintended behavioural responses by encouraging couples to stay childless for longer (Abramowitz, 2014 & 2017; Rainer et al., 2011; Gershoni & Low, 2021), hence increasing their risk of experiencing infertility and of underachieving their reproductive plans. This is especially relevant when considering the widespread misconceptions in the population regarding the biological limits to reproduction and the chances of achieving conception and birth at later ages via ART (Pedro et al., 2018).

Third, the higher incidence of multiple births following ART deliveries inflates the estimated contribution of these procedures to fertility rates. Though the rate of multiple births following ART cycles has decreased in Australia, from a peak of 22.1% in 2000 (Dean & Sullivan, 2003) to 2.9% in 2017 (Newman et al., 2021), it is still higher than the rate of multiple deliveries from all conceptions (1.5%) (Australian Institute of Health and Welfare, 2019b). Hence, especially among older cohorts, the contribution of ART to CFR is partly attributable to the higher incidence of multiple births. Ultimately, the magnitude to which multiple births bias our results depends on the extent to which couples using ART wish to have only one or more than one child. For the latter group, a multiple pregnancy is contributing to the faster achievement of the desired family size and, hence, does not imply an overestimation of births.

Fourth, the data and methods used do not allow for a more detailed analysis of the relationship between fertility postponement, ART fertility, and non-ART fertility. Arguably, the main factor in this interaction is the ongoing shift to later parenthood, which reduces the number of naturally conceived births due to infertility and increases the demand for and use of ART. More subtle mechanisms and interactions also play a role, as conceptualized in Fig. 2. For instance, improved chances of ART conception can boost ART fertility, increase demand for treatments, and thus suppress non-ART fertility rates at younger ages. Couples in successive cohorts may be more willing to use ART, which then lowers the chances of achieving a non-ART pregnancy and birth. In addition, attitudes towards ART may change over time and alter the relationship between ART fertility and fertility postponement. Properly modeling these interactions would require detailed assumptions about fertility preferences, demand for ART, numbers of cycles used, and ‘natural’ (non-ART) fertility rates among women from different cohorts, which is beyond the scope of our study. However, we indirectly account for the impact of delayed parenthood on ART births in scenarios S3–S5, which assume an ongoing increase in the use of ART.

Another limitation lies in the implicit assumption of our extrapolation method of linear growth in ART treatment and success rates. Linear trends may not always be accurate, as events such as the COVID-19 pandemic can disrupt the provision and uptake of ART treatment in unexpected ways (Rodriguez-Wallberg & Wikander, 2020). Although the adoption of a cohort approach reduces the bias introduced by changes in fertility timing, other shocks and technological changes may be more difficult to anticipate. For instance, it is plausible that ART success rates may increase more sharply in the future because of the introduction of new treatments and advancements in reproductive technology. Aware of these uncertainties, our projections adopt a conservative approach by limiting trend extrapolation to a five-year forecast horizon, taking into account the potential for unforseen changes in demand for ART, technology, and childbearing postponement.

## Discussion

Using data from a comprehensive clinical registry of ART treatments and births, we projected the contribution of ART to completed fertility in Australia under five alternative scenarios. The percentage contribution of ART to completed fertility is estimated to increase from 2.1% among women born in 1968 to between 4.7% and 5.7% among women born in 1986. The relative contribution of ART will more than double between the analysed cohorts even in the most conservative *No-change scenario* (S1). The probability of having an ART-conceived child increases with age and across cohorts. In the youngest cohorts (1980–1986), approximately one in three children born to women aged 45–49 and one in four children born to women aged 40–44 are expected to be conceived via ART. Our findings are robust across scenarios and the adoption of a cohort approach implies that results are not sensitive to short-term changes in treatment rates and success rates introduced by temporary shocks, including temporary disruption to ART provision during the recent COVID-19 pandemic. Even in the most conservative *No-change scenario* (S1) in which we assumed that there will be no further increases in the demand for ART and no improvements in the effectiveness of treatments after the last observed year, 2017, the contribution of ART is substantial and increasing due to the continuation of the trend towards childbearing postponement among the youngest cohorts.

The in-depth analysis of the contribution of ART to fertility by age adopting a cohort approach is essential for evaluating fertility trends as well as fertility ‘recuperation’ at later ages in low fertility countries. While most past research focused on the impact of ART on overall fertility (Habbema et al., 2009; Hoorens et al., 2007; Leridon, 2017; Leridon & Slama, 2008; Sobotka et al., 2008), our results demonstrate that ART substantially contributes to fertility at older reproductive ages and corroborate previous research demonstrating the crucial role played by ART in increasing period fertility rates past the age of 40 (Lazzari et al., 2021).

Our scenarios suggest that increases in ART fertility rates will be mainly driven by an increase in the demand for infertility treatment. However, the diffusion of alternative treatment options, such as egg freezing and the use of third-party donor eggs, may lead to a sharp rise in ART success rates and alter these dynamics in the future. Our study also explored the extent to which postponed childbearing will be compensated at older ages due to ART. Compared to a hypothetical situation where treatment is not available, the fertility recuperation among cohorts of women currently in their early to mid-30s (born between 1982 and 1986) is twice as large when ART is considered. Remarkably, further fertility recovery after age 40 is almost entirely attributable to ART-conceived births.

ART is constantly advancing. New methods may further improve success rates and increase the number of ART births beyond our projected trends Biological relatedness is important to many people, which suggests that oocyte cryopreservation may be more widely acceptable than donor methods (Shreffler et al., 2010). The growing number of women freezing their eggs supports this idea (Johnston et al., 2021), although elective egg freezing remains a controversial technology, with high psychological and monetary costs^3^ for prospective mothers (Jackson, 2018).

This study has a broader relevance for demographers and policymakers, as it sheds light on the potential role of ART for future fertility. The growing use of ART reflects broader changes in family formation in low fertility countries, which include a long-term shift of parenthood to older reproductive ages (Beaujouan, 2020; Mills et al., 2011). More women are having children past the age of 30 due to delays in partnership and marriage, as well as more frequent second and third partnerships after breakups and separations (Thomson et al., 2014). Because of the biological constraints on fertility, ART births are likely to make up an increasing proportion of children. Hence, the general finding on the important role of ART in fertility recuperation highlighted by this study is likely to hold across all the countries experiencing the postponement transition. Such contribution can be leveraged especially in settings where there is no upper age limit set by law to access treatments, like in Australia. In countries with different ART funding arrangements and regulations, the overall contribution of treatments to cohort fertility will likely vary. Hence, the results from this study can be more easily generalizable to settings with generous public systems for infertility treatment and high ART utilization rates. These conditions are met especially in the Nordic countries (Wyns et al., 2020).

As childbearing before the age of 30 continues to decline, a compensatory increase in fertility rates at later ages will become crucial for supporting future fertility levels. Advances in reproductive technologies have likely pushed the upper age limit of fertility to new extremes, as noted by Billari et al., (2007, p. 166). However, an effort to empirically analyse the impact of ART on late and very late fertility is only recently emerging in the demographic literature. Our study contributes to this research area by demonstrating that ART may become an important contextual driver of fertility recovery.

## Supplementary Information

Below is the link to the electronic supplementary material.

Supplementary file1 (DOCX 152 kb)

## Data Availability

The data on which this article is based cannot be shared publicly for confidentiality reasons.

## Footnotes

1 Own computations based on Australian Bureau of Statistics (ABS) data.

2 This study adopts a deterministic approach to predict future cohort fertility and the likely contribution of ART. In our model, uncertainty is incorporated through the elaboration of alternative scenarios about future conditions affecting the number, share, and rate of ART births among the analysed cohorts.

3 Egg freezing is an expensive medical procedure even in countries with a supportive funding environment for ART like Australia because it is not typically covered by Medicare and by any other national insurance schemes since it is not considered clinically necessary.
