# Supplementary material for: Projecting the Contribution of Assisted Reproductive Technology to Completed Cohort Fertility
Source: Popul Res Policy Rev. 2023 Feb 10;42(1):6. doi: 10.1007/s11113-023-09765-3 (PMC9912242; doi:10.1007/s11113-023-09765-3)
Supplement: Supplementary file 1 — Supplementary file1 (DOCX 152 kb) [file 11113_2023_9765_MOESM1_ESM.docx]

**Supplementary Material**

**Contents:**

1. Data sources
2. Linear five-year extrapolation method
3. Robustness check – Adding education
4. Differences between scenarios
5. **Data sources**

**
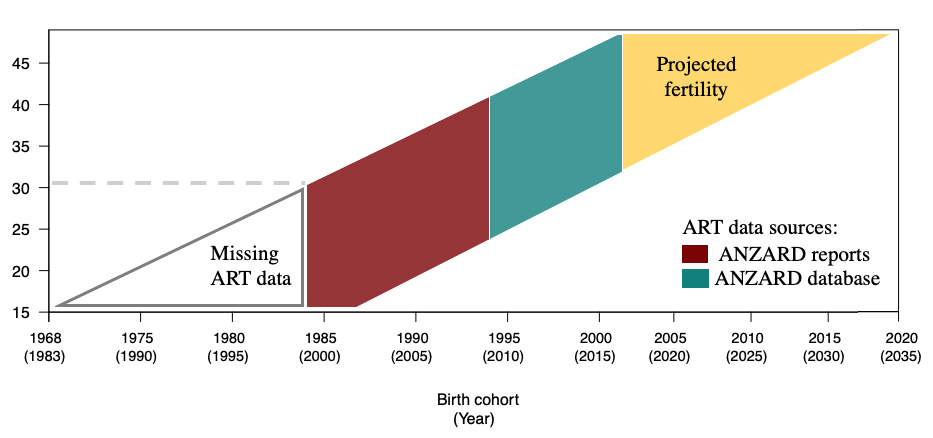
**

**Fig S1**. Lexis surface showing a representation of the data sources and analyzed birth cohorts.

1. **Linear 5-year extrapolation method**

The extrapolation model forecasts age-specific fertility rates $f\left( x,t \right)$ for age $x$ at time $t$ as:

$f\left( x,t \right)= a\left( x \right)+b\left( x \right)K(t)$ (s1)

Where $a\left( x \right)$ is the baseline age-specific fertility rate and fertility change is decomposed into a time component $K\left( t \right)$and age component $b\left( x \right)$. To forecast fertility, $K\left( t \right)$is extrapolated with a random walk with drift δ:

$K\left( t \right)= K\left( t-1 \right)+\partial+\epsilon(t)$ (s1)

Where ϵ(t) is the error term.

For a detailed description of the method, see Myrskylä et al. (2013).

1. **Robustness check - Adding education**

The projected ART age-specific fertility rates for the period 2017-2022 were first estimated for women with low, medium and high education, and then aggregated to produce total ART fertility rates based on the educational composition of the individual cohorts. Below we describe the data and methodological approach used to estimate ART fertility histories by education and report the results of such analysis.

*Data Sources*

Education-specific treatment rates are estimated using an indirect estimation method, consisting of the following steps: a) calculation of the proportion of women of parity 0 and parity 1 and above by educational group from the census; b) distribution of ART treatments across educational groups based on parity information; c) calculation of treatment rates by education; d) estimation of ART ASFR by education obtained by multiplying treatment rates by success rates. Since there is no evidence that ART success rates vary by education, the same success rates have been used for all educational groups. Total assisted ASFRs are obtained by: 1) splitting each cohort of women in three groups according to their educational attainment in 2016, and 2) assigning to each group the estimated education-specific ART fertility pattern. Because the use of ART is more common among first-born children and highly educated women make up the largest share of childless women at all ages above 30, a higher proportion of ART births is assigned to tertiary educated women (Figure S2).

[Fig. S2 here]

The 2016 Australian Census of Population and Housing (Census) microdata, accessed through TableBuilder Pro (Australian Bureau of Statistics, 2021b), are used to assess the educational composition of the cohorts of women born in 1968-1986. Hence, information about women’s educational attainment corresponds to their educational level in 2016, when they were aged between 30 and 48 years old. These estimates largely reflect their level of education at the end of their reproductive life, as the majority of women has completed their education by age 30. Educational attainment is separated into three educational groups^[[1]](#footnote-1)^: low for women with primary and lower secondary education; medium for women with upper- and post-secondary education; and high for women with any tertiary degree obtained through university. In Figure S3, the proportion of the population with low, medium and high education are presented for 1968-1986 cohorts. A substantial increase in the proportion of women with tertiary degrees took place among the analysed cohorts, jumping from fewer than 30% to 45%. The second piece of information needed to estimate education-specific ART fertility rates are the proportions of childless women and of women with one or more children by educational group, also sourced from the 2016 Census (Australian Bureau of Statistics, 2021b). These data allow us to create a connection between records in the ANZARD database and in the 2016 Census based on parity information and to redistribute ART births across low, medium and high educated women.

[Fig. S3 here]

*Results*

Overall, the adoption of this alternative method that considers educational composition does not substantially alter our results and confirms the robustness of our findings. Results are presented in table S1.

[Table S1]

**Fig. S2**. ART age-specific treatment rates, 2016, Australia.

Note: Educational attainment was measured using three categories, based on the Australian Standard Classification of Education (ASCED): low for women with certificate and Year 11 or below qualifications; medium for women with Year 12 and diploma qualifications; and high for women with any tertiary degree obtained through university.

Source: Authors’ computations based on the 2016 Australian Census of Population and Housing microdata and ANZARD data.

**
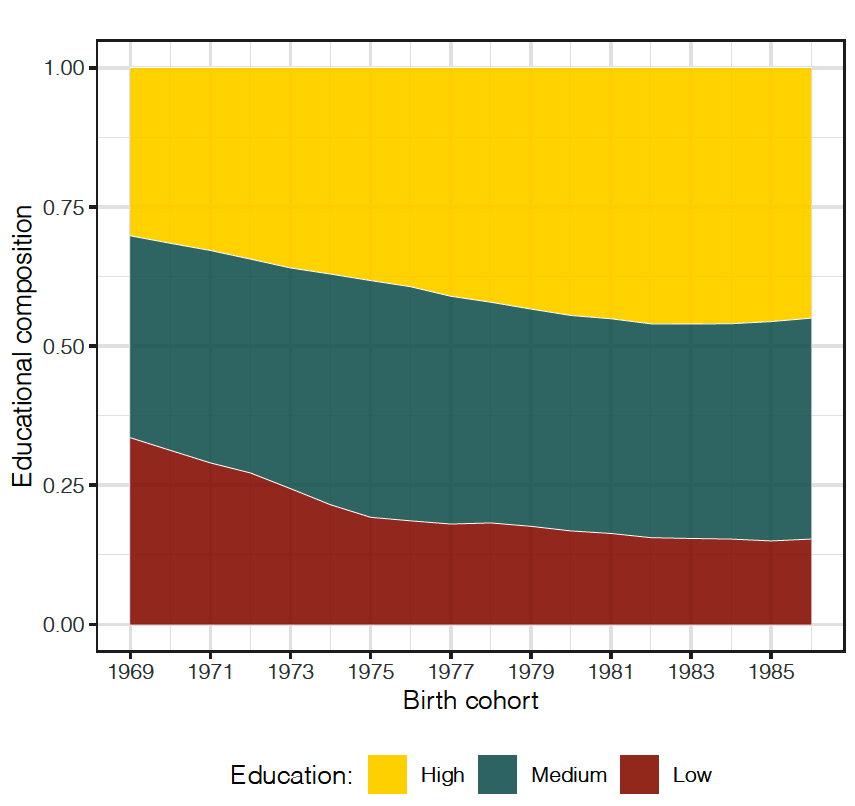
**

**Fig. S3**. Educational composition among women born in 1968-1986, Australia.

Note: Educational attainment was measured using three categories, based on the Australian Standard Classification of Education (ASCED): low for women with certificate and Year 11 or below qualifications; medium for women with Year 12 and diploma qualifications; and high for women with any tertiary degree obtained through university.

Source: Authors’ computations based on the 2016 Australian Census of Population and Housing microdata.

**Table S2**. Estimated percentage effect of ART use on the CFR according to five scenarios, Australian women born 1968-1986.

| Birth cohort | Scenario 1 | Scenario 2 | Scenario 3 | Scenario 4 | Scenario 5 |
| --- | --- | --- | --- | --- | --- |
| 1968 | 2.07 | 2.07 | 2.07 | 2.07 | 2.07 |
| 1969 | 2.26 | 2.26 | 2.26 | 2.26 | 2.26 |
| 1970 | 2.48 | 2.48 | 2.48 | 2.49 | 2.49 |
| 1971 | 2.69 | 2.70 | 2.70 | 2.70 | 2.71 |
| 1972 | 2.90 | 2.91 | 2.91 | 2.92 | 2.94 |
| 1973 | 3.14 | 3.16 | 3.15 | 3.18 | 3.22 |
| 1974 | 3.40 | 3.43 | 3.42 | 3.45 | 3.56 |
| 1975 | 3.65 | 3.69 | 3.68 | 3.73 | 3.90 |
| 1976 | 3.82 | 3.87 | 3.86 | 3.92 | 4.18 |
| 1977 | 3.95 | 4.00 | 4.00 | 4.08 | 4.39 |
| 1978 | 4.07 | 4.14 | 4.16 | 4.25 | 4.60 |
| 1979 | 4.18 | 4.28 | 4.31 | 4.44 | 4.77 |
| 1980 | 4.32 | 4.42 | 4.50 | 4.63 | 4.96 |
| 1981 | 4.39 | 4.50 | 4.62 | 4.76 | 5.10 |
| 1982 | 4.42 | 4.52 | 4.72 | 4.85 | 5.19 |
| 1983 | 4.44 | 4.53 | 4.79 | 4.92 | 5.26 |
| 1984 | 4.58 | 4.68 | 5.01 | 5.14 | 5.48 |
| 1985 | 4.62 | 4.71 | 5.10 | 5.22 | 5.56 |
| 1986 | 4.67 | 4.75 | 5.21 | 5.31 | 5.66 |

Source: Authors’ computations based on ANZARD and ABS data.

1. **Differences between scenarios**

**Fig. S4**. Observed (1968) and projected (1969-1986) completed cohort fertility, selected scenarios.

Source: Authors’ computations based on ANZARD and ABS data.

**Table S3.** Estimated percentage effect of ART use on the CFR according to five scenarios, Australian women born 1968-1986.

| Birth cohort | S1 (%) | Relative % increase as compared to S1 | | | |
| --- | --- | --- | --- | --- | --- |
|  |  | (S2 – S1) | (S3 – S1) | (S4-S1) | (S5-S1) |
| 1968 | 2.07 | 0.00 | 0.00 | 0.00 | 0.00 |
| 1969 | 2.26 | 0.00 | 0.00 | 0.00 | 0.00 |
| 1970 | 2.49 | 0.00 | 0.00 | 0.00 | 0.01 |
| 1971 | 2.73 | 0.00 | 0.00 | 0.01 | 0.01 |
| 1972 | 2.91 | 0.01 | 0.01 | 0.02 | 0.02 |
| 1973 | 3.15 | 0.02 | 0.01 | 0.03 | 0.06 |
| 1974 | 3.42 | 0.03 | 0.02 | 0.05 | 0.12 |
| 1975 | 3.68 | 0.04 | 0.03 | 0.08 | 0.20 |
| 1976 | 3.88 | 0.05 | 0.04 | 0.10 | 0.28 |
| 1977 | 4.05 | 0.05 | 0.06 | 0.13 | 0.35 |
| 1978 | 4.18 | 0.07 | 0.09 | 0.18 | 0.39 |
| 1979 | 4.30 | 0.10 | 0.12 | 0.25 | 0.45 |
| 1980 | 4.47 | 0.10 | 0.17 | 0.30 | 0.50 |
| 1981 | 4.55 | 0.11 | 0.23 | 0.37 | 0.56 |
| 1982 | 4.61 | 0.10 | 0.29 | 0.42 | 0.61 |
| 1983 | 4.64 | 0.09 | 0.35 | 0.48 | 0.67 |
| 1984 | 4.76 | 0.10 | 0.42 | 0.55 | 0.74 |
| 1985 | 4.81 | 0.08 | 0.48 | 0.59 | 0.78 |
| 1986 | 4.85 | 0.07 | 0.53 | 0.64 | 0.83 |

Note: S2-5 show the difference in the estimated percentage effect of ART use as compared to S1.

No-change Scenario (S1)

Extrapolated success rates Scenario (S2)

Extrapolated treatment rates Scenario (S3)

Extrapolated success and treatment rates Scenario (S4)

Egg donation and freezing Scenario (S5)

Source: Authors’ computations based on ANZARD and ABS data.

1. The classification of education follows the Australian Standard Classification of Education (ASCED): low for women with certificate and Year 11 or below qualifications, medium for women with Year 12 and diploma qualifications, and high for women with Bachelor, Master and Doctoral degrees. [↑](#footnote-ref-1)
